# Supplementary material for: Visible Light Reductive Photocatalysis of Azo-Dyes with n–n Junctions Based on Chemically Deposited CdS
Source: Molecules. 2022 May 4;27(9):2924. doi: 10.3390/molecules27092924 (PMC9105509; doi:10.3390/molecules27092924)
Supplement: Supplementary file 1 [file molecules-27-02924-s001.zip › molecules-1682815-supplementary.pdf]

Supplementary Materials

Visible Light Reductive Photocatalysis of Azo-Dyes with n–n Junctions Based on Chemically Deposited CdS

Michele Mazzanti, Martina Milani, Vito Cristino, Rita Boaretto, Alessandra Molinari * and Stefano Caramori *

Dipartimento di Scienze Chimiche, Farmaceutiche ed Agrarie, Università di Ferrara, Via Luigi Borsari 46, 44121 Ferrara, Italy; michele.mazzanti@unife.it (M.M.); martina.milani@unife.it (M.M.); vito.cristino@unife.it (V.C.); rita.boaretto@unife.it (R.B.)

* Correspondence: alessandra.molinari@unife.it (A.M.); stefano.caramori@unife.it (S.C.)

Table of Contents

Figure S1. Profilometry

Figure S2. AFM 3D map, top view and size analysis of FTO/ZrO_2/CdS and FTO/TiO_2/CdS

Figure S3. XRD patterns of FTO/CdS, FTO/ZrO_2/CdS and FTO/TiO_2/CdS

Figure S4. Tauc Plots of FTO/ZrO_2 /CdS and FTO/TiO_2/CdS

Figure S5. Emission spectrum of FTO/CdS, FTO/ZrO_2/CdS and FTO/TiO_2/CdS

Figure S6. Emission decays of FTO/ZrO_2/CdS

Figure S7. Emission decays of FTO/TiO_2/CdS

Figure S8. Dark Cyclic voltammetry of the FTO/CdS

Figure S9. Dark Cyclic voltammetry of the FTO/TiO_2/CdS

Figure S10. Dark Cyclic voltammetry of the FTO/ZrO_2/CdS

Figure S11. Open circuit chronopotentiometry

Figure S12. Cyclic voltammetry of MO, AO7 and EDS

Figure S13. ESI-MS spectra of MO and EDS after irradiation with FTO/TiO_2/CdS

Figure S14. C/C_0 *vs* irradiation time of azo-dyes with FTO/ZrO_2/CdS and ESI-MS spectrum of EDS after irradiation

Table S1. Kinetic constant and half time values determined by dyes concentration decrease under visible light irradiation with FTO/MO_2/CdS systems.

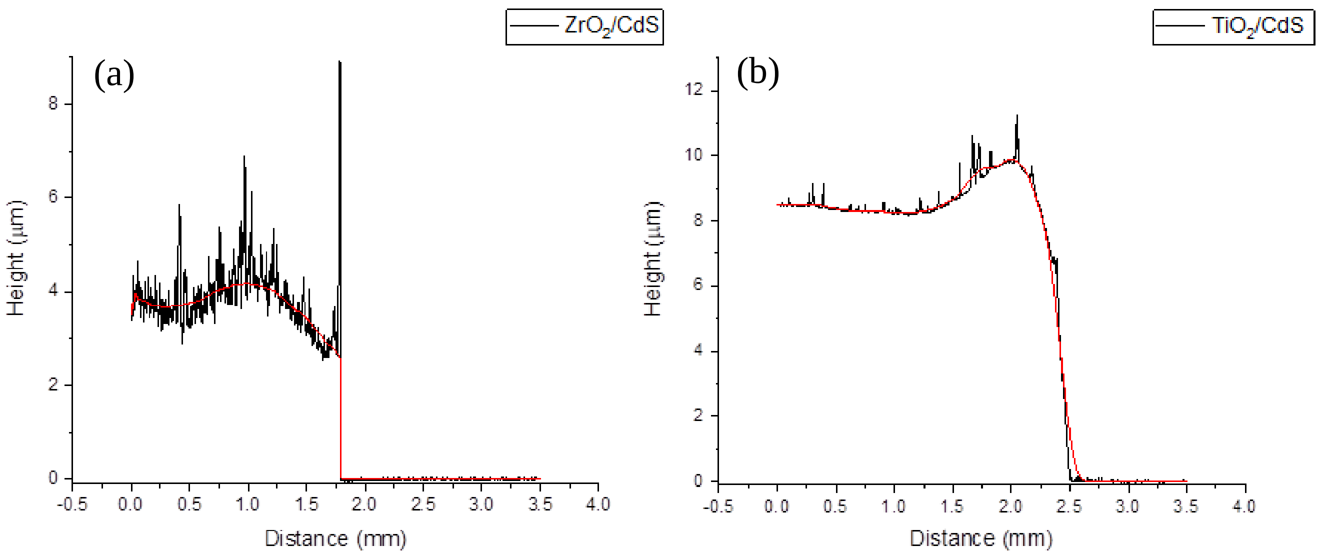

Figure S1. Thickness measurements of (**a**) FTO/ZrO_2/CdS and (**b**) FTO/TiO_2/CdS

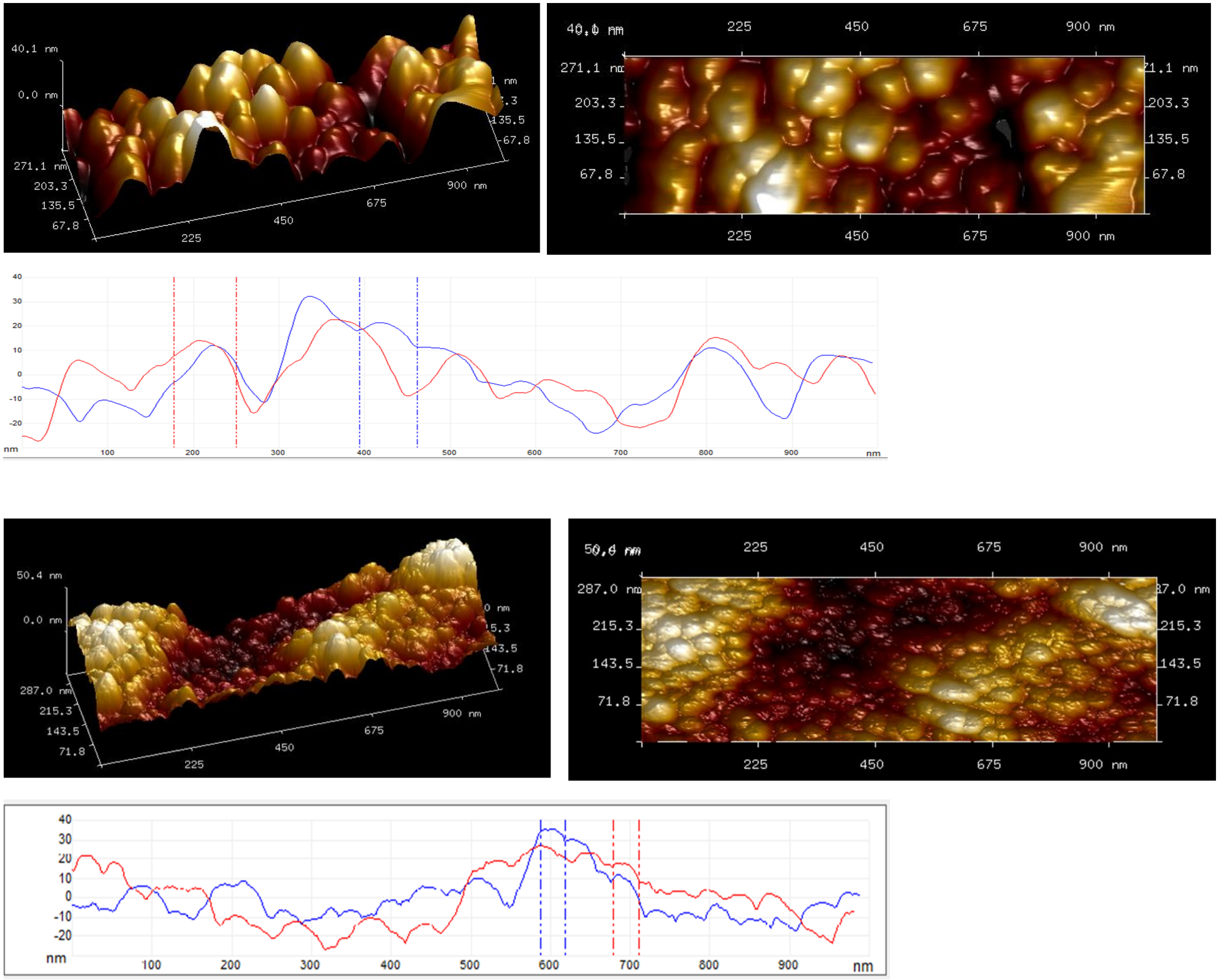

Figure S2. AFM 3D map, top view and cross sectional size analysis of (**a**) $FTO/TiO_2/CdS$ and (**b**) $FTO/ZrO_2/CdS$

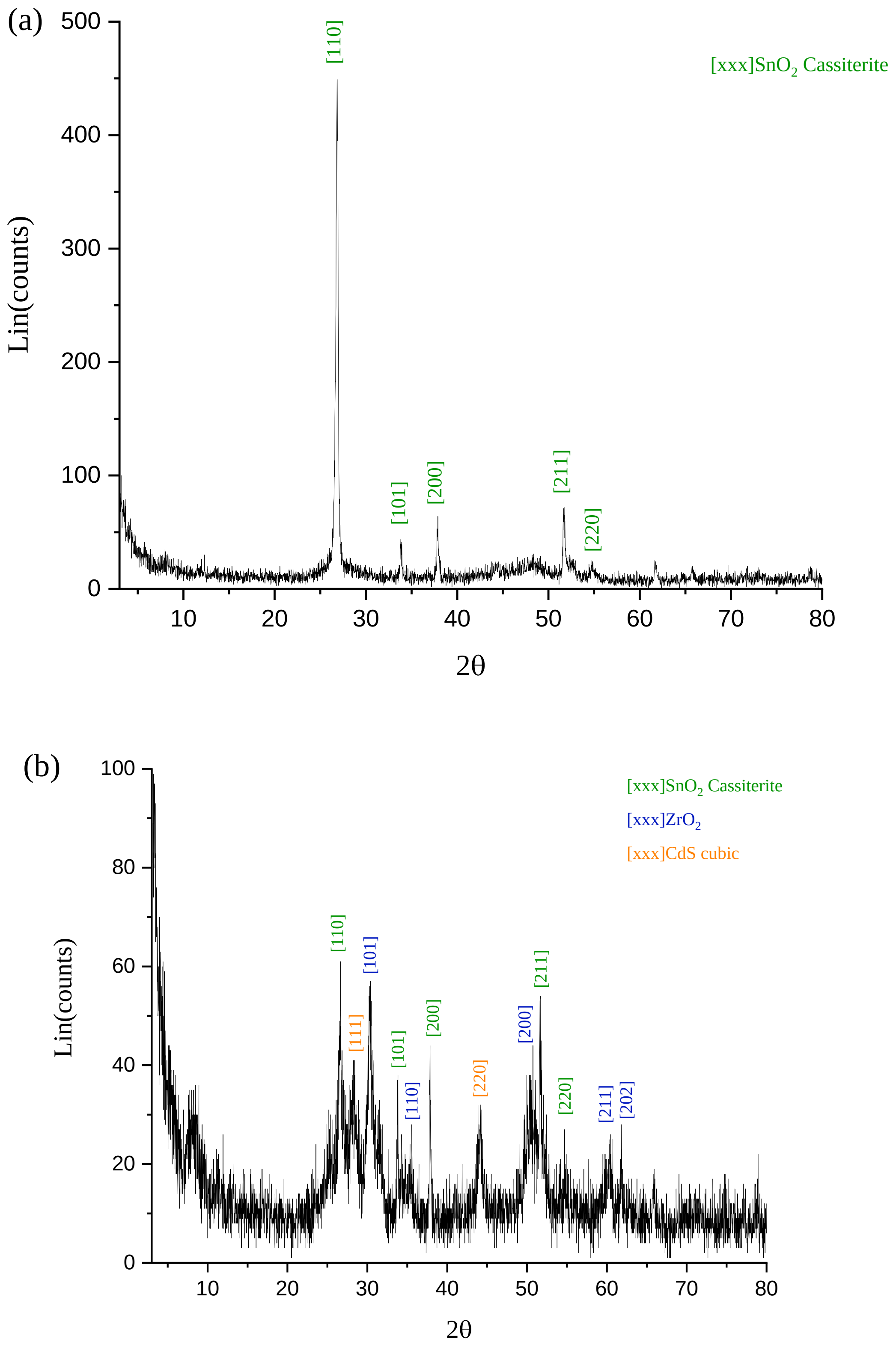

(a)
[xxx]SnO_2 Cassiterite
[110]
[101]
[200]
[211]
[220]
Lin(counts)
2θ
0
100
200
300
400
500
10
20
30
40
50
60
70
80
(b)
[xxx]SnO_2 Cassiterite
[xxx]ZrO_2
[xxx]CdS cubic
[110]
[101]
[111]
[101]
[110]
[200]
[220]
[200]
[211]
[220]
[211]
[202]
Lin(counts)
2θ
0
20
40
60
80
100
10
20
30
40
50
60
70
80

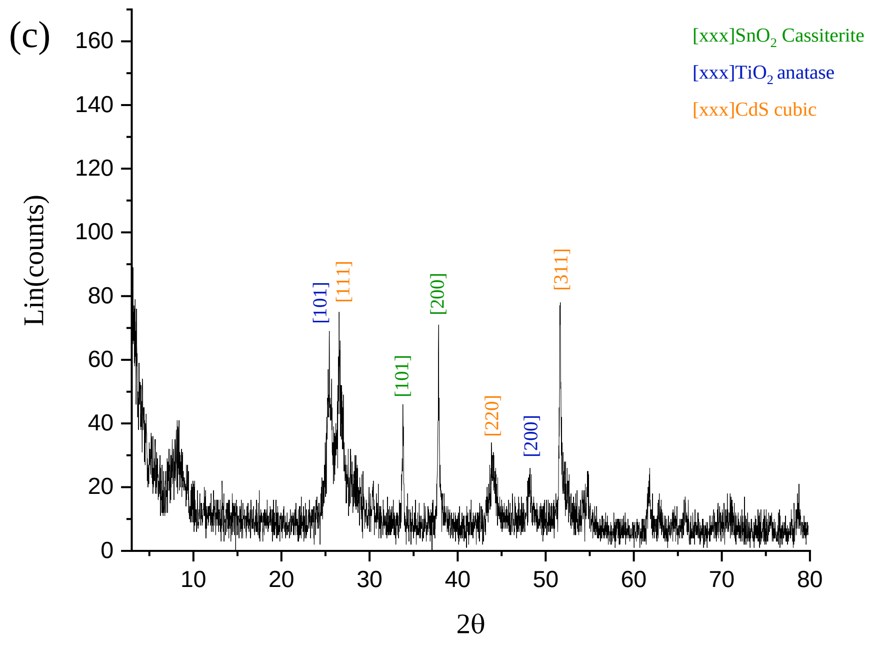

Figure S3. XRD patterns of (**a**) FTO/CdS, (**b**) FTO/ZrO_2/CdS (**c**) FTO/TiO_2/CdS

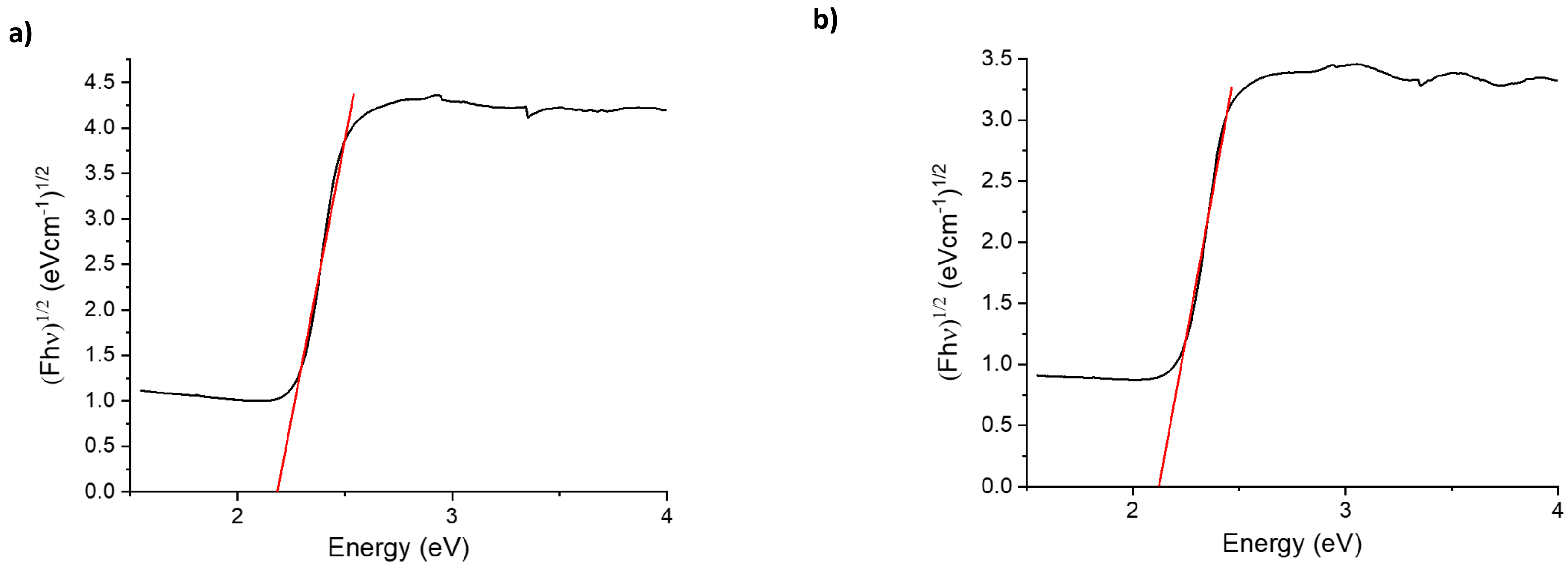

Figure S4. Tauc Plots of (a) FTO/ZrO_2 /CdS and (b) FTO/TiO_2/CdS

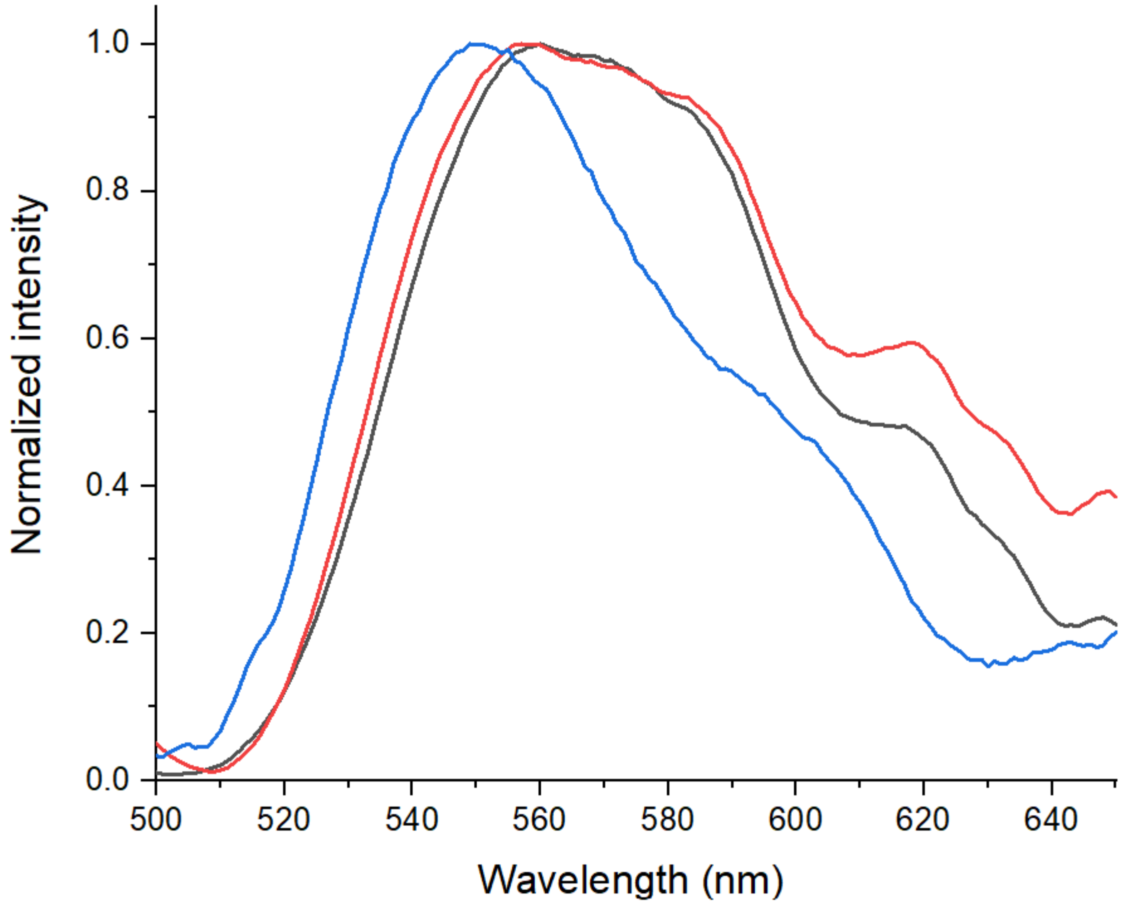

Figure S5. Emission spectra (λ_{exc} = 450 nm) of FTO/CdS, FTO/ZrO_2/CdS, FTO/TiO_2/CdS thin films at room temperature.

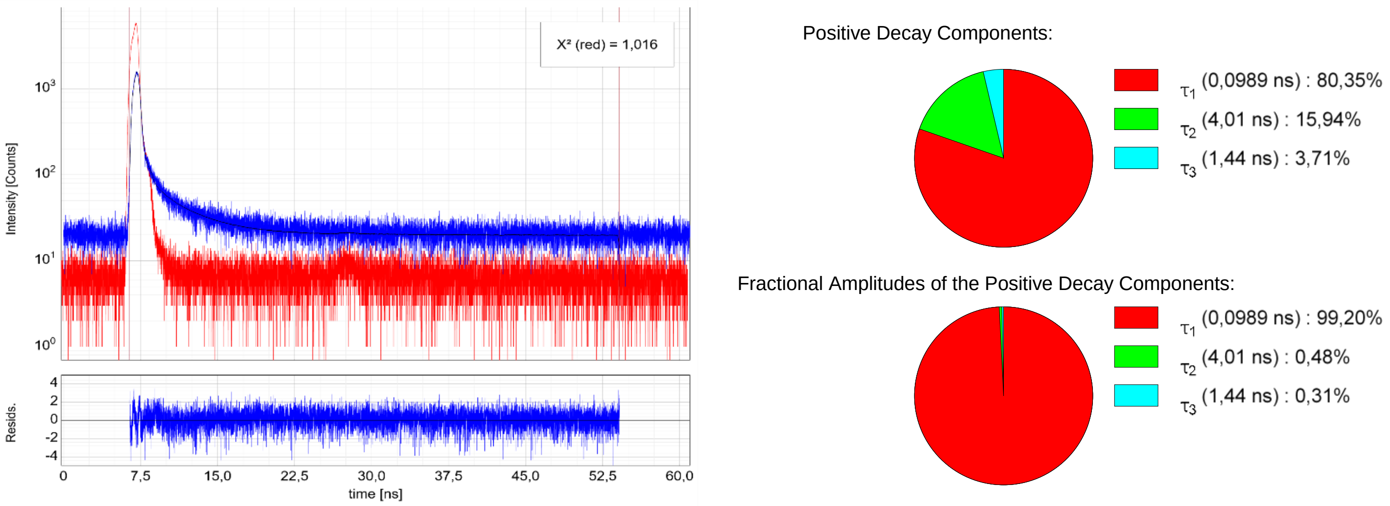

Parameter	Value	Conf. Lower	Conf. Upper	Conf. Estimation
A_1 [Cnts]	2312	-50	+50	Fitting
τ1 [ns]	0,0989	-0,0021	+0,0021	Fitting
A_2 [Cnts]	11,30	-0,96	+0,96	Fitting
τ2 [ns]	4,01	-0,31	+0,31	Fitting
A_3 [Cnts]	7,3	-2,3	+2,3	Fitting
τ3 [ns]	1,44	-0,47	+0,47	Fitting
Bkgr. Dec [Cnts]	-6,83	-0,81	+0,81	Fitting
Bkgr. IRF [Cnts]	-67,3	-2,2	+2,2	Fitting
Shift IRF [ns]	-0,209	-0,010	+0,010	Fitting
A Scat [Cnts]	-57900	-7200	+7200	Fitting
Period Rep [ns]	0,0587	-0,0048	+0,0048	Fitting

Figure S6. Single photon timing emission decay of FTO/ZrO_2/CdS in 1M sodium formate

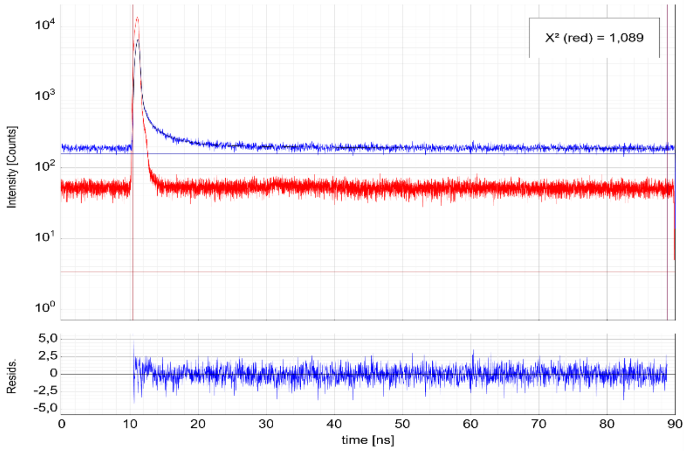

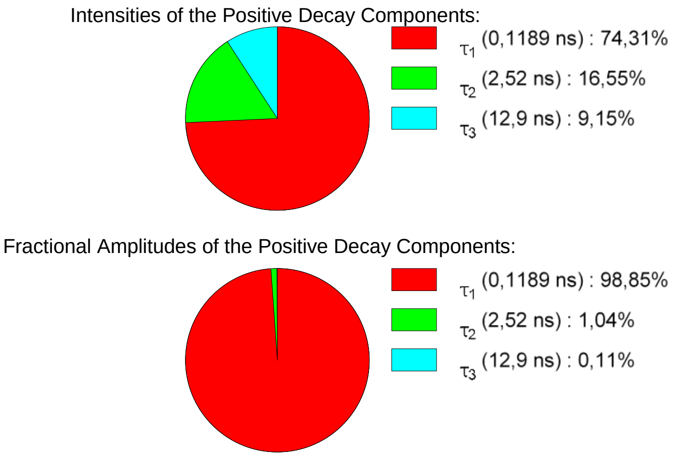

Parameter	Value	Conf. $_{Lower}$	Conf. $_{Upper}$	Conf. $_{Estimation}$
A_1 [Cnts]	8150	-190	+190	Fitting
τ1 [ns]	0,1189	-0,0027	+0,0027	Fitting
A_2 [Cnts]	85,7	-7,5	+7,5	Fitting
τ2 [ns]	2,52	-0,20	+0,20	Fitting
A_3 [Cnts]	9,3	-2,0	+2,0	Fitting
τ3 [ns]	12,9	-2,9	+2,9	Fitting
Bkgr. $_{Dec}$ [Cnts]	158,9	-2,4	+2,4	Fitting
Bkgr. $_{IRF}$ [Cnts]	3,4	-3,7	+3,7	Fitting
Shift $_{IRF}$ [ns]	-0,1421	-0,0077	+0,0077	Fitting
A $_{Scat}$ [Cnts]	145000	-37000	+37000	Fitting
Period $_{Rep}$ [ns]	-0,00026	-0,0071	+0,0071	Fitting

Figure S7. Single photon timing emission decay of FTO/TiO_2/CdS in 1M sodium formate

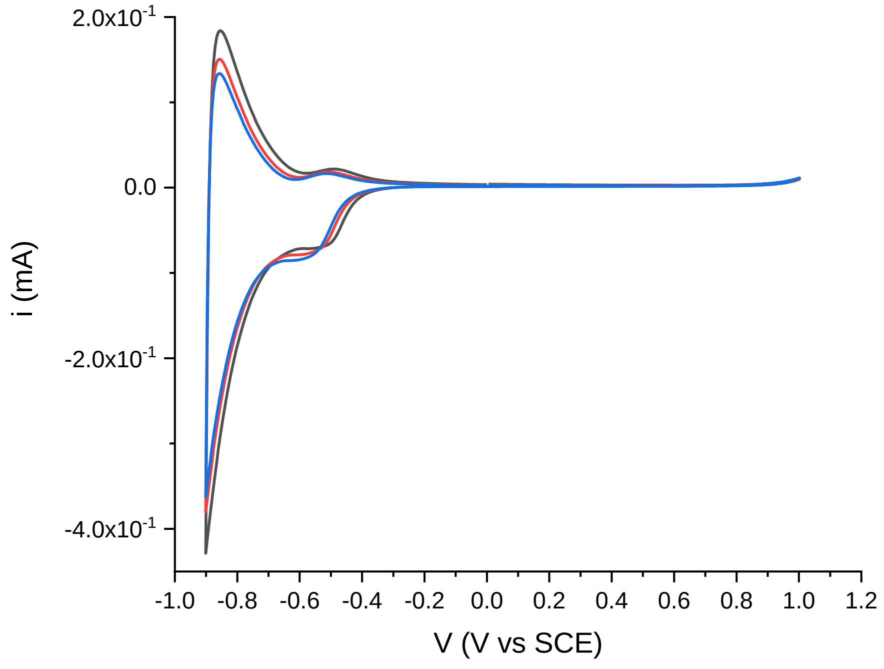

Figure S8. Dark cyclic voltammetry of the FTO/TiO_2/CdS recorded in HCOONa 1M at pH 7 between -1V/1V vs SCE at a scan rate of 50mV/s

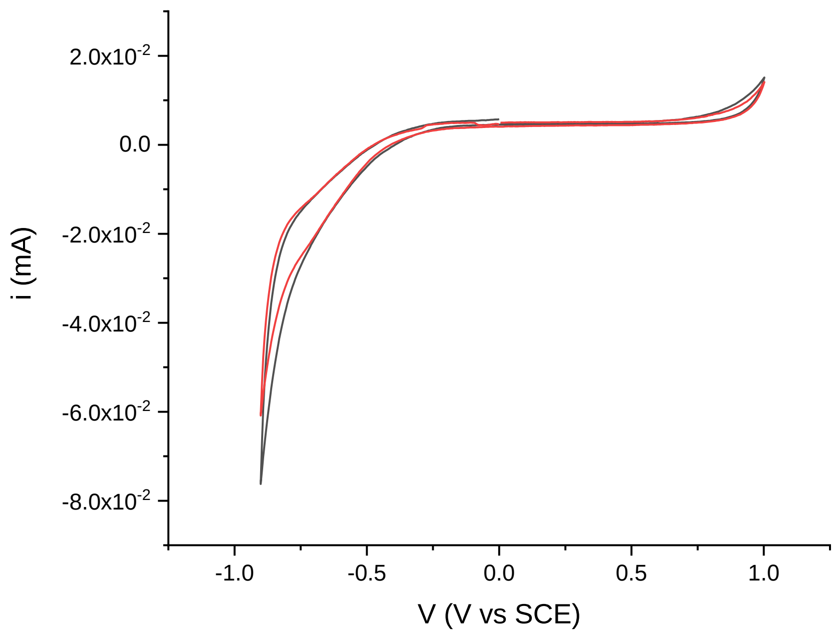

Figure S9. Dark cyclic voltammetry of the FTO/CdS recorded in HCOONa 1M at pH 7 between -1V/1V vs SCE at a scan rate of 50mV/s

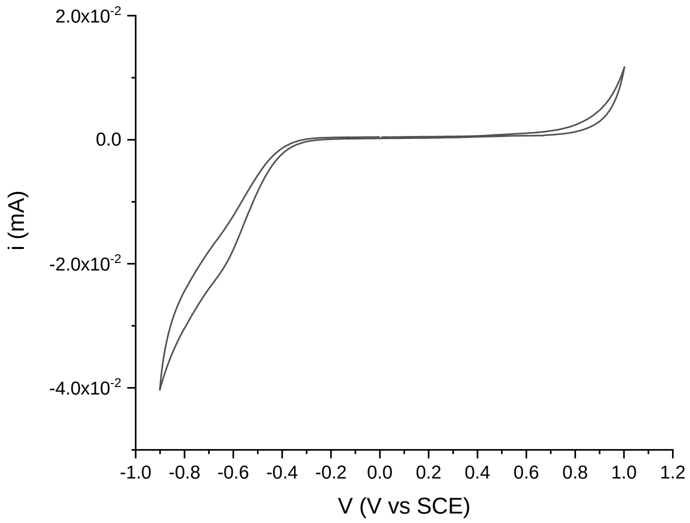

Figure S10. Dark cyclic voltammetry of the FTO/ZrO_2/CdS recorded in HCOONa 1M at pH 7 between -1V/1V vs SCE at a scan rate of 50mV/s

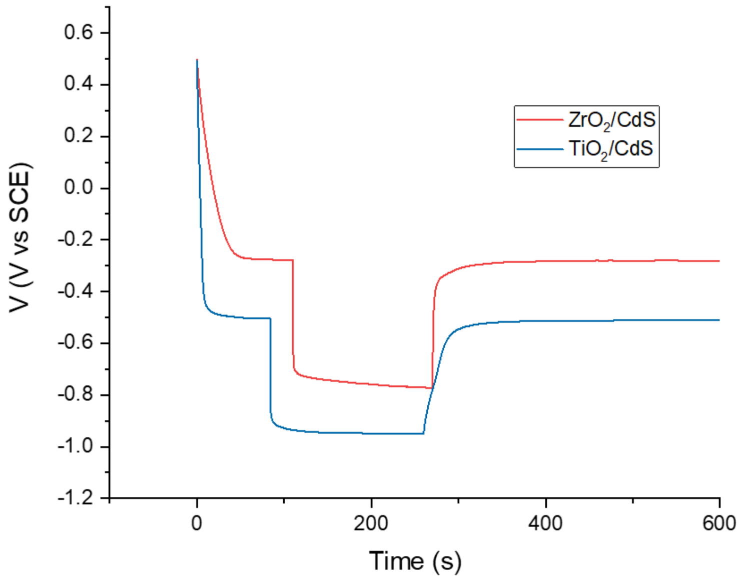

Figure S11. Open circuit chronopotentiometry of FTO/ZrO_2/CdS (red) and FTO/TiO_2/CdS (blue) in the presence of 1 M HCOONa at pH 7.

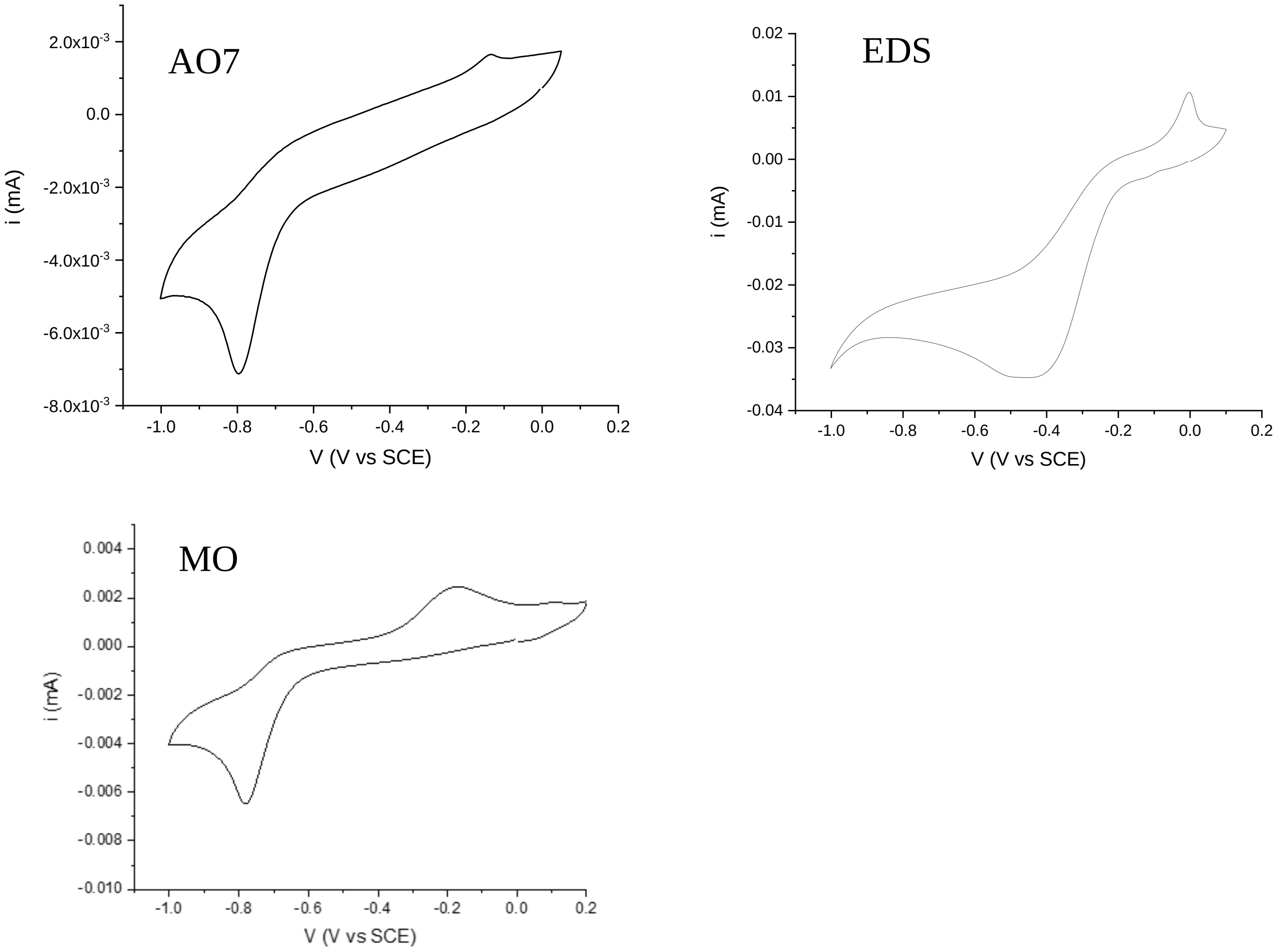

Figure S12. Cyclic voltammetry of the MO, AO7 and EDS dyes (20 mV/s) recorded in the presence of 0.1 M $LiClO_4$ at a glassy carbon electrode.

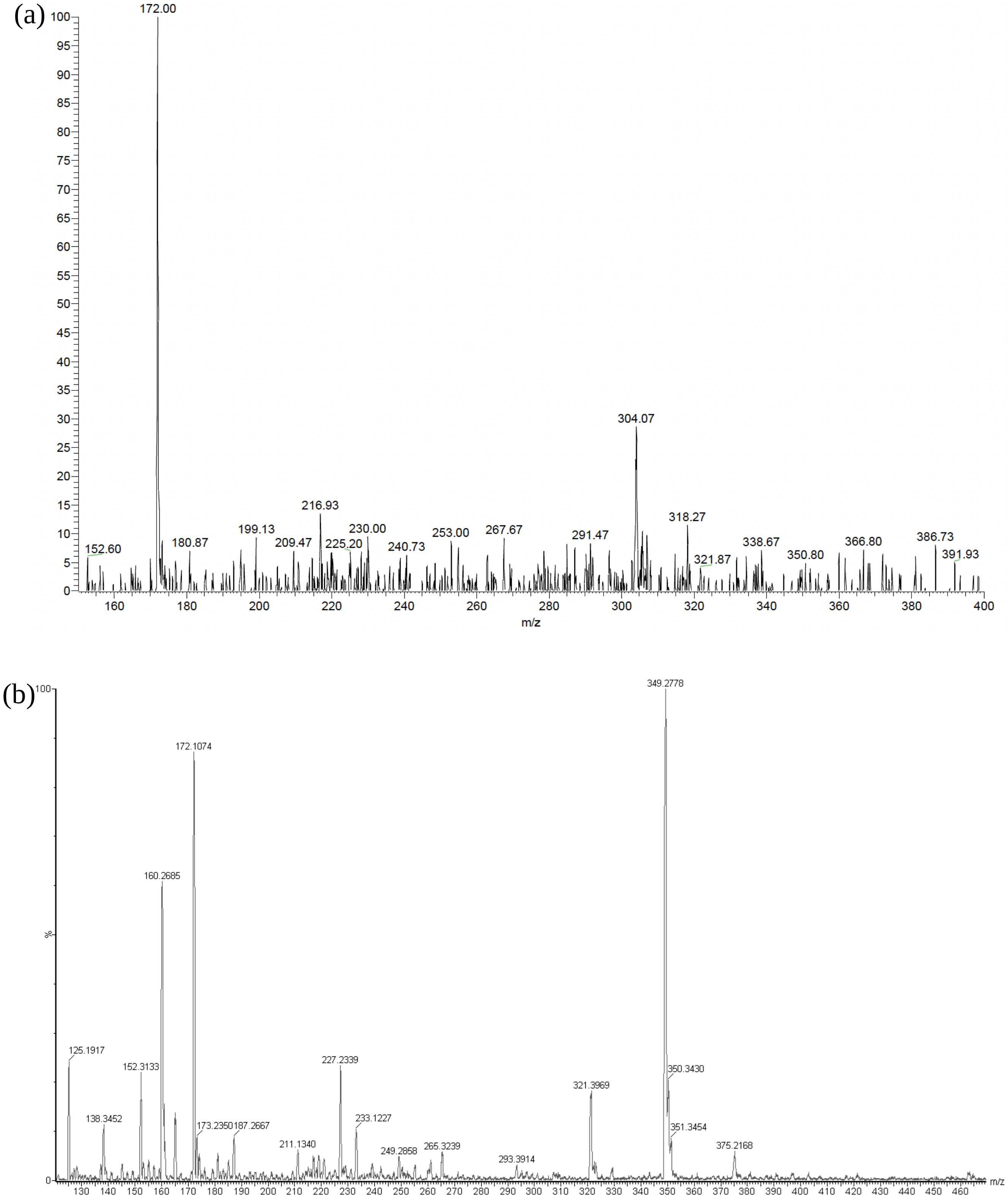

Figure S13. (**a**): ESI-MS spectrum of the deaerated aqueous solution containing MO (10 ppm) and ethanol (10% v/v) after irradiation ($\lambda \geq 420$ nm) in the presence of FTO/TiO_2/CdS system. (**b**) the same as part a but with EDS (10 ppm). Ethanol is used here in place of formate as a nonionic hole scavenger.

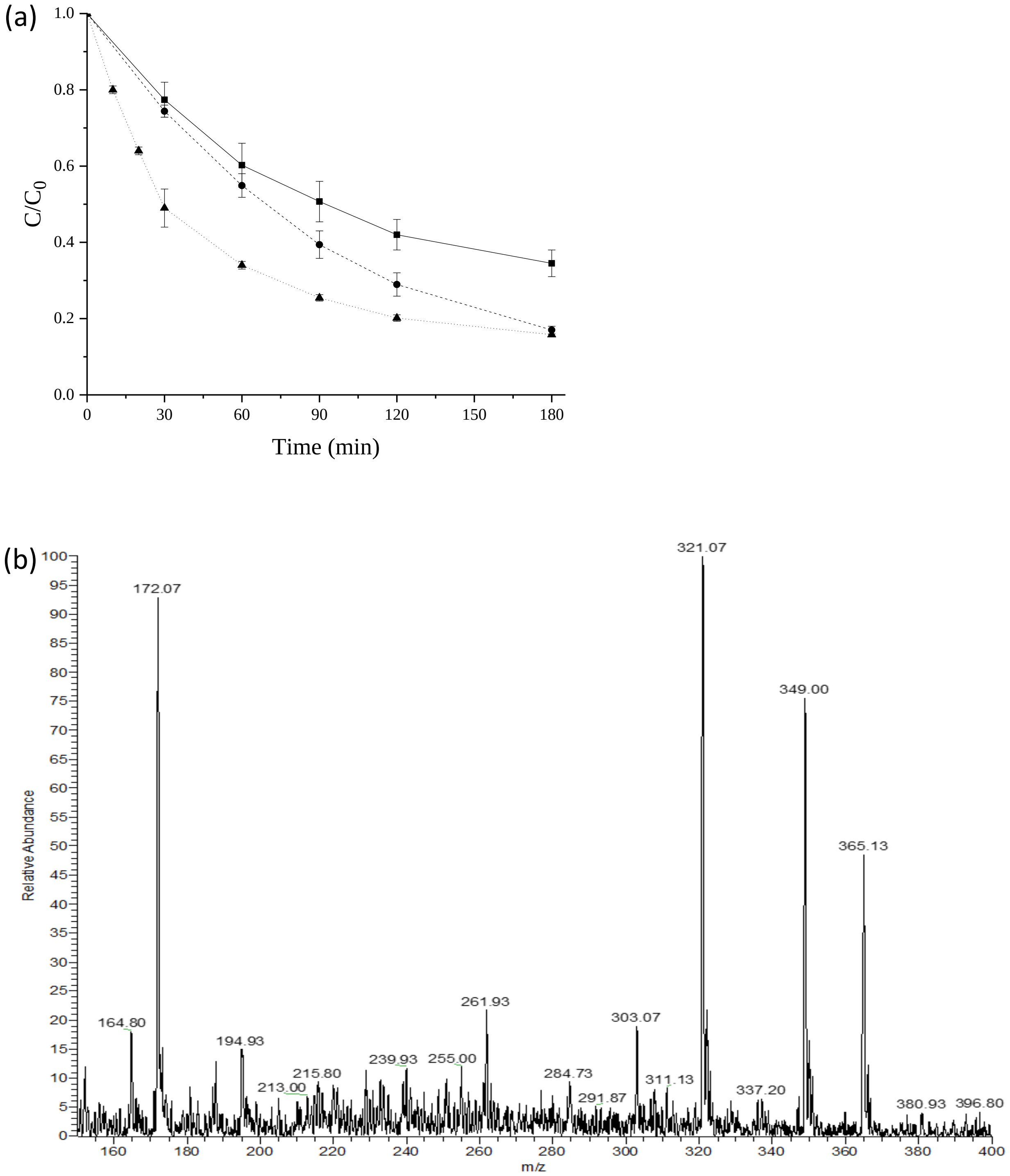

Figure S14. (**a**) C/C_0 ratio vs. time profiles obtained upon irradiation ($\lambda \geq$ 420 nm) of FTO/ZrO_2/CdS film immersed in deaerated aqueous solutions containing HCOONa (1 M) and MO (circle), or AO7 (triangle), or EDS (square). C_0 = 10 ppm. Reported data are the mean of three repeated experiments. (**b**) ESI-MS spectrum of deaerated aqueous solution containing EDS (10 ppm) and ethanol (10% v/v) after irradiation (λ > 420 nm) in the presence of FTO/ZrO_2/CdS system

Table S1. Kinetic constants and half-time values determined by dyes concentration decrease under visible light irradiation with FTO/MO_2/CdS systems.

	Dye	*k (min^{-1})*	$t_{1/2}$	R^2
FTO/TiO_2/CdS	MO	0.0125	55	0.9977
	AO7	0.0283	24	0.9726
	EDS	0.0123	56	0.9004
FTO/ZrO_2/CdS	MO	0.0104	66	0.9933
	AO7	0.0178	39	0.9407
	EDS	0.0072	96	0.9711
